# The Comparison of Tree-Sibling Time Consistent Phylogenetic Networks Is Graph Isomorphism-Complete

**DOI:** 10.1155/2014/254279

**Published:** 2014-04-02

**Authors:** Gabriel Cardona, Mercè Llabrés, Francesc Rosselló, Gabriel Valiente

**Affiliations:** ^1^Department of Mathematics and Computer Science, University of the Balearic Islands, 07122 Palma de Mallorca, Spain; ^2^Algorithms, Bioinformatics, Complexity and Formal Methods Research Group, Technical University of Catalonia, 08034 Barcelona, Spain

## Abstract

Several polynomial time computable metrics on the class of semibinary tree-sibling time consistent phylogenetic networks are available in the literature; in particular, the problem of deciding if two networks of this kind are isomorphic is in P. In this paper, we show that if we remove the semibinarity condition, then the problem becomes much harder. More precisely, we prove that the isomorphism problem for generic tree-sibling time consistent phylogenetic networks is polynomially equivalent to the graph isomorphism problem. Since the latter is believed not to belong to P, the chances are that it is impossible to define a metric on the class of all tree-sibling time consistent phylogenetic networks that can be computed in polynomial time.

## 1. Introduction

After the realization that reticulation processes, like hybridizations, recombinations, or lateral gene transfers, have been more relevant in the evolution of life on Earth than previously thought [1], there has been a growing interest in the development of algorithms for the reconstruction of* phylogenetic networks*: graphical models of evolutionary histories that go beyond phylogenetic trees by including* hybrid nodes* of in-degree greater than one representing reticulation events. As the number of available such algorithms increases, the need of methods for the comparison of phylogenetic networks also increases, as they are used, for instance, to assess the reliability and robustness of these algorithms [2, 3].

One of the types of phylogenetic networks for which there exist reconstruction methods [4, 5] are the* tree-sibling time consistent* networks,* TSTC networks*, for short (see [6] for a formal definition). Two metrics on the class of all* semibinary* TSTC networks, where all hybrid nodes have in-degree two, have been proposed in the last years. Both metrics are based on encodings of phylogenetic networks that turn out to single out any TSTC network among all such networks: the *μ-vectors* (where each node in the network is represented by the vector of numbers of paths from it to each leaf) [7] and the* nested labels* (where each node in the network is represented by a certain Newick-like representation of the subnetwork rooted at it) [8, 9]. Actually, this last metric turns out to be sound also for the class of all semibinary tree-sibling networks, without the time-consistency restriction [6].

But, although there have been several attempts to define a metric on the class of all TSTC networks on a given set of taxa [10], none of the metrics for phylogenetic networks computable in polynomial time proposed so far satisfies the separation axiom (distance 0 means isomorphism) for TSTC networks [8, 11, 12]. In this paper we show why it should come as no surprise; such a metric would solve in polynomial time the graph isomorphism problem.

The graph isomorphism problem is one of the most important decision problems for which the computational complexity is not known yet [13, 14]. It is believed to be neither in P nor NP complete, and subexponential time solutions for it are known. A problem is said to be* graph isomorphism-complete* when it is polynomially equivalent to the graph isomorphism problem [15]. In this paper we show that, for every set *S* with more than two elements, the isomorphism problem for TSTC phylogenetic networks with taxa bijectively labeled in *S* is graph isomorphism-complete.

## 2. Preliminaries

Let *G* = (*V*, *E*) be a nonempty rooted directed acyclic graph (a rDAG, for short). A node of *G* is a* leaf* if it has out-degree 0,* internal* if its out-degree is ⩾1, of* tree* type if its in-degree is ⩽1, of* hybrid* type if its in-degree is >1, and* elementary* if it is a tree node of out-degree 1. A node *v* is a* child* of another node *u* (and, hence, *u* is a* parent* of *v*) if (*u*, *v*) ∈ *E*. Two nodes *u* and *v* are* siblings* of each other if they share a parent. An arc (*u*, *v*) in a rDAG is a* tree arc* when *v* is a tree node and a* hybridization arc* when *v* is a hybrid node. The* height* of a node *v* is the longest length of a directed path from *v* to a leaf, and the* depth* of *v* is the longest length of a directed path from the root to *v*.

Given a finite set *S* of* labels*, a *S-*rDAG is a rDAG with its leaves injectively labelled by *S*. By an isomorphism of *S*-rDAGs we understand an isomorphism of directed graphs that preserves and reflects the labelling, that is, that matches each leaf in one network with the leaf with the same label in the other network. In a *S*-rDAG, we will always identify without any further reference every leaf with its label.

A* phylogenetic network* on a set *S* of* taxa* is a *S*-rDAG such that

- no tree node is elementary;
- every hybrid node has out-degree 1, and its single child is a tree node.

A* phylogenetic tree* is a phylogenetic network without hybrid nodes.

We will say that a phylogenetic network is* tree-sibling* if every hybrid node has at least one sibling that is a tree node.

A* temporal assignment* [16] on a network *N* = (*V*, *E*) is mapping *τ* : *V* → *ℕ* such that

- if *v* is a hybrid node and (*u*, *v*) ∈ *E*, then *τ*(*u*) = *τ*(*v*);
- if *v* is a tree node and (*u*, *v*) ∈ *E*, then *τ*(*u*) < *τ*(*v*).

We will say that a phylogenetic network is* time-consistent* if it admits a temporal assignment. The following alternative characterization of time consistency will be used later. For a proof, see [16, 17].

**Proposition 1**

Let *N* = (*V*, *E*) be a phylogenetic network, let *E*_*H*_ be its set of hybridization arcs, and let N* = (*V*, E*) be the directed graph with the same set *V* of nodes as *N* and set of arcs E* = *E* ∪ {(*v*, *u*) | (*u*, *v*) ∈ *E*_*H*_}. Then, *N* is time consistent if, and only if, N* does not have any cycle containing some tree arc of *N*.

For short, we will refer henceforth to tree-sibling time consistent phylogenetic networks simply as* TSTC networks*.

The underlying biological motivation for the definitions on phylogenetic networks introduced so far is the following. In a phylogenetic network, tree nodes model species (either extant, the leaves, or nonextant, the internal tree nodes), while hybrid nodes model reticulation events, where different species interact to create new species. The parents of the hybrid node represent the species involved in this event and its single child being the resulting species. The tree children of a tree node represent direct descendants through mutation. The first condition in the definition of phylogenetic network says that every nonextant species is assumed to have at least two different direct descendants. This is a very common restriction in any definition of phylogeny (be it a tree or a network), since species with only one child cannot be reconstructed from biological data.

The tree-sibling condition says then that, for every reticulation event, at least one of the species involved in it must have some descendant through mutation. This condition was introduced with the name* class I* in L. Nakhleh's Ph.D. thesis [10], and it has reappeared in several phylogenetic network reconstruction methods [4, 5]. As far as the time consistency goes, we understand that the time assigned to a node represents the time when the corresponding species existed, or when the reticulation event took place. The first condition in time consistency means then that the species involved in a reticulation event must coexist in time in order to interact, while the second condition means that speciation takes some amount of time to take place.

## 3. Main Results

It is well known [13, 18] that the isomorphism problem for rDAGs is graph isomorphism-complete. It turns out that the isomorphism problem for rDAGs with their leaves injectively labeled in any given set of labels is also graph isomorphism-complete; since we have not been able to find a proof of this easy result in the literature, we provide one here.

**Proposition 2**

For every nonempty set *S* of labels, the isomorphism for *S*-rDAGs is graph isomorphism-complete.

**Proof**

Without any loss of generality, we assume that *S* = {1,…, *n*}⊆*ℕ*.

**Figure 1:**
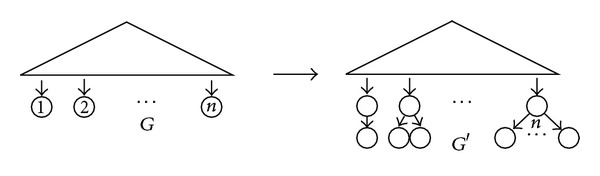
The construction involved in the reduction of the isomorphism of *S*-rDAGs to the isomorphism of rDAGs.

Let us prove first that the isomorphism of *S*-rDAGs reduces to the isomorphism of rDAGs. For every *S*-rDAG *G*, let *G*′ be the rDAG obtained from *G* by unlabelling its leaves and, then, for each *k* = 1,…, *n*, if *G* contained a leaf labeled with *k*, then adding to this leaf *k* tree-children leaves; see Figure 1. The construction of *G*′ from *G* = (*V*, *E*) adds *O*(*n* ^2^) ⩽ *O*(|*V*|^2^) nodes and arcs, and therefore it is polynomial in the size of *G*. And *G* can be reconstructed from *G*′ by simply replacing, for each *k* = 1,…, *n*, the node of height 1 with *k* leaves by a leaf labeled with *k*. Then, it is straightforward to check that, for every pair of *S*-rDAGs *G*_1_ and *G*_2_ over *S*, *G*_1_≅*G*_2_ as *S*-rDAGs if, and only if, *G*_1_′≅*G*_2_′ as rDAGs.

**Figure 2:**
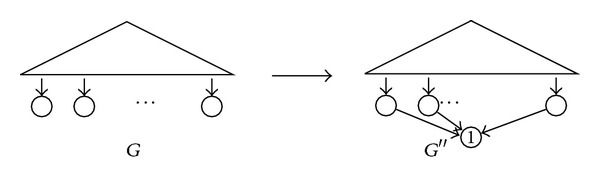
The construction involved in the reduction of the isomorphism of rDAGs to the isomorphism of *S*-rDAGs.

Let us prove now that the isomorphism of rDAGs reduces to the isomorphism of *S*-rDAGs. For every rDAG *G*, let *G*′′ be the *S*-rDAG obtained from *G* by adding a new node *a*, arcs from each leaf of *G* to *a*, and finally labeling the new node *a* with 1; see Figure 2. The construction of *G*′′ from *G* = (*V*, *E*) adds 1 node and *O*(|*V*|) arcs, and therefore it is polynomial. And *G* can be reconstructed from *G*′′ by simply removing its leaf and all arcs pointing to it. It is straightforward to check that, for every pair of rDAGs *G*_1_ and *G*_2_ over *S*, *G*_1_≅*G*_2_ if, and only if, *G*_1_′≅*G*_2_′ as *S*-rDAGs.

Let us see now that the isomorphism problem for *S*-rDAGs reduces to the isomorphism problem for TSTC networks on a new set of labels consisting of *S* and two extra labels. This entails that the isomorphism of TSTC networks on sets with at least three labels is graph isomorphism-complete.

**Theorem 3**

For every set *S* with |*S* | ⩾3, the isomorphism of TSTC networks on *S* is graph isomorphism-complete.

**Proof**

Without any loss of generality, we assume that *S* = {1,…, *n*}⊆*ℕ*.

The isomorphism of TSTC networks on *S* clearly reduces to the isomorphism of *S*-rDAGs, since the former is a special case of the latter. Let us prove now the converse reduction.

We will associate to each *S*-rDAG *N* = (*V*, *E*) a TSTC network $\bar{N}$ on *S* ∪ {*n* + 1, *n* + 2}. If *N* is a phylogenetic tree, then it is already a TSTC network, and in this case we take $\bar{N}=N$. Consider now the case when *N* has some hybrid node or some elementary node, and let *m* be the largest label actually appearing in *N*. In this case, we define the TSTC network $\bar{N}$ as follows.

1. For every hybrid node *h* in *N*, remove all arcs from *h* to its children, and then add a new (tree) node *u*_*h*_, an arc from *h* to *u*_*h*_, and new arcs from *u*_*h*_ to the children of *h* in *N*. If *h* was a leaf, say with label *k*, then *u*_*h*_ becomes the new leaf labeled with *k*.
2. For every hybridization arc *e* = (*v*, *h*) in the resulting *S*-rDAG, split it into arcs (*v*, *v*_*e*_) and (*v*_*e*_, *h*), with *v*_*e*_ a new (tree and, for the moment, elementary) node. Let *N*′ denote the resulting *S*-rDAG after these two first steps.
3. For every elementary node *v* in *N*, add a new (tree) node *v*′ and an arc (*v*, *v*′).
4. Split the arc (*w*, *m*) in *N*′ pointing to the leaf *m* into two arcs (*w*, *w*_*m*_) and (*w*_*m*_, *m*).
5. Add two new nodes *a* and *b*, and, for every node *v*′ added in step (3), add arcs (*v*′, *a*) and (*v*′, *b*). Add also arcs (*w*_*m*_, *a*) and (*w*_*m*_, *b*). Notice that the nodes *a* and *b* will be hybrid.
6. Add a tree leaf children labelled *n* + 1 to *a* and another one labelled *n* + 2 to *b*.

**Figure 3:**
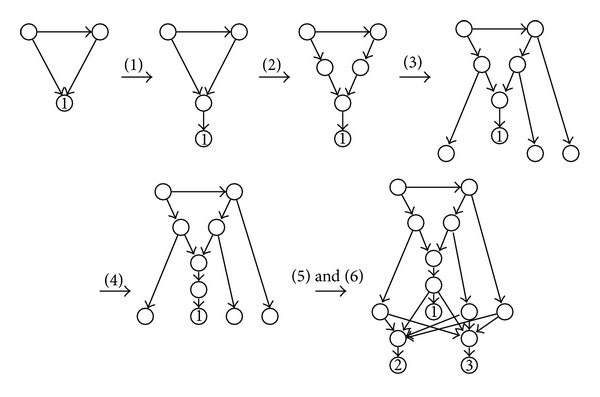
An example of the construction involved in the reduction of the isomorphism of *S*-rDAGs to the isomorphism of TSTC networks.

An example of this construction is displayed in Figure 3.

Let us prove now that $\bar{N}$ is a tree-sibling time consistent phylogenetic network.

- It is rooted (with the same root as *N*) and acyclic, because all new arcs are either used to split arcs in *N* into pairs of consecutive arcs, or to define paths that end in the new leaves *n* + 1 or *n* + 2 without forming cycles.
- It has no elementary node. Indeed, any elementary node in *N* gets an extra child in step (3), and the tree nodes that are added to *N* either get an extra child in step (3) or they get two children in (5).
- Its hybrid nodes have only one child, and it is a tree node; this is ensured for the hybrid nodes in *N* in step (1), and for the new hybrid nodes *a* and *b* by construction.
- It is tree-sibling. All hybrid nodes in *N* get a tree sibling in steps (2) and (3) (for every hybrid node *h* in *N*, if *e* is any arc pointing to *h*, then the tree child *v*_*e*_′ of the new node *v*_*e*_ added in the middle of *e* is such a tree sibling of *h*), and the hybrid nodes *a* and *b* have the tree sibling *m*.
- It is time consistent. To check this, we use Proposition 1 (and the notations introduced therein). Since we already know that $\bar{N}$ is acyclic, any cycle in ${\bar{N}}^{*}$ must contain some inverse of a hybridization arc. There are two possibilities for this inverse. If it has the form (*h*, *x*), with *h* one of the new hybrid nodes *a* or *b* introduced in step (5) and *x* one of the tree nodes *v*′ introduced in step (3) or the tree node *w*_*m*_ introduced in step (4), then the only tree arcs that can be reached from *x* in ${\bar{N}}^{*}$ are those pointing to the leaves *m*, *n* + 1 or *n* + 2, and therefore no cycle in ${\bar{N}}^{*}$ contains this arc (*h*, *x*) together with a tree arc. And if this inverse is of the form (*h*, *v*_*e*_), with *h* a hybrid node in *N* and *v*_*e*_ one of the tree nodes introduced in step (2), then it must be followed in the cycle by the arc (*v*_*e*_, *v*_*e*_′) added in step (3), and, as we have just said, the only tree arcs that can be reached from *v*_*e*_′ point to a leaf, and hence no cycle in ${\bar{N}}^{*}$ contains this arc (*h*, *v*_*e*_′) and a tree arc, either.

It is clear that the construction of $\bar{N}$ from *N* adds *O*(|*V* | +|*E*|) nodes and arcs to *N*, and thus it is polynomial in the size of *N*. Notice also that in this case $\bar{N}$ always contains hybrid nodes, and in particular that it is never a phylogenetic tree. Moreover, in this nontree case, the *S*-rDAG *N* can be easily reproduced from $\bar{N}$ by simply undoing its construction as follows.

1. Remove the leaves *n* + 1 and *n* + 2 and their hybrid parents *a* and *b*, together with all arcs pointing to them.
2. Remove the elementary parent of the leaf *m* (which will be the remaining leaf with largest label in *S*) and replace it by an arc from the parent of the removed node to *m*.
3. Remove all nonlabeled leaves of the resulting rDAG together with the arcs pointing to them.
4. Remove each parent *v*_*e*_ of every hybrid node, and replace it by an arc from the parent of *v*_*e*_ to the hybrid child of *v*_*e*_.
5. Remove the only tree child of each hybrid node, and replace it by an arc from the hybrid node to each one of the children of the removed node.
6. The resulting *S*-rDAG is *N*.

It is straightforward to check now that, for every pair of *S*-rDAGs *N*_1_ and *N*_2_, *N*_1_≅*N*_2_ if, and only if, $\bar{N_{1}}≅\bar{N_{2}}$ as phylogenetic networks over *S* ∪ {*n* + 1, *n* + 2}.

We cannot remove the condition |*S* | ⩾3 in the previous result because there are only two TSTC networks with less than 3 leaves (up to the actual names of the labels). In particular, this implies that, in the proof of the previous result, we cannot add less than 2 new leaves in the construction of $\bar{N}$ from *N*.

**Proposition 4**

There is only one TSTC network with one leaf, and only one TSTC phylogenetic with two leaves (up to relabeling), and in both cases they are trees.

**Proof**

The {1}-rDAG consisting of a single node, labeled 1, and the {1,2}-rDAG consisting of the phylogenetic tree with Newick code (1,2); are clearly TSTC networks. Let us check now that any other (up to relabeling) TSTC network has at least 3 leaves.

Let *N* = (*V*, *E*) be a TSTC network other than those described in the last paragraph, let *τ* : *V* → *ℕ* be a time assignment, and let *v* be an internal node with largest *τ*-value and, among those with this largest time assignment, of largest depth.

If *v* is a tree node, then all its children are either leaves or hybrid nodes with leaf children (because any tree descendant node of *v* has time assignment larger than *τ*(*v*)). And *v*'s hybrid children would have the same time assignment as *v* but depth largest than *v*'s depth, against the assumption. Therefore all children of *v* are leaves, and it has at least 2 children, because it cannot be elementary. Now, if *v* has more than 2 children, we are done, while if it has only two children, say the leaves 1 and 2, then *v* will have a parent in *N* (because *N* is not the tree (1,2);). If the parent of *v* is a tree node, let *w* be this node, and let *z* be another child of *w*. Since *N* does not contain cycles, and any path to 1 or 2 must contain *w*, we deduce that any descendant leaf of *z* must be different from 1 or 2; this gives at least 3 leaves. If, on the contrary, the parent of *v* is a hybrid node *x*, let *w* be the parent of *x* that has a tree child, say *z*. The time consistency prevents *x* to be a descendant of *z* (because *τ*(*z*) > *τ*(*w*) = *τ*(*x*)) and, therefore, since any path leading to 1 or 2 must contain *x*, any leaf that is a descendant of *z* will be different from 1,2; this gives again at least 3 leaves.

If *v* is a hybrid node, then its child is a leaf, say 1. Let *v*_1_ be a parent of *v* that has a tree child. Since *τ*(*v*_1_) = *τ*(*v*) is the largest *τ* value of an internal node of *N*, this tree child must be a leaf, say 2. Now let *v*_2_ be another parent of *v*. Since it is a tree node, it must have another child other than *v*, say *x*. If *x* is a tree node, it is a leaf, as we have just seen. If *x* is hybrid, then since *τ*(*x*) = *τ*(*v*_2_) = *τ*(*v*), the tree child of *x* must be a leaf. In both cases, we obtain a leaf that is different from 1 and 2; that is, *N* contains at least 3 leaves.

It is usual in the literature to define a phylogenetic network on a set *S* of taxa as an rDAG with its leaves bijectively labeled in *S*.  Theorem 3 also holds in this case.

**Corollary 5**

For every set *S* with |*S*|⩾3, the isomorphism of TSTC networks with leaves bijectively labeled on *S* is graph isomorphism-complete.

**Proof**

The isomorphism of TSTC networks with leaves bijectively labeled on *S* clearly reduces to the isomorphism of TSTC networks with leaves injectively labeled on *S*, since the former is a special case of the latter. For the converse reduction, let *N*_1_ and *N*_2_ be two TSTC networks with leaves injectively labeled on *S*, let *S*_1_⊆*S* be the leaf labels of *N*_1_, and let *S*_2_⊆*S* be the leaf labels of *N*_2_. If *S*_1_ ≠ *S*_2_, then *N*_1_ and *N*_2_ are not isomorphic. If *S*_1_ = *S*_2_, let ${\bar{N}}_{1}$ and ${\bar{N}}_{2}$ be the TSTC networks obtained by adding to the roots of *N*_1_ and *N*_2_, respectively, |*S*∖*S*_1_| leaf children bijectively labeled on *S*∖*S*_1_. These TSTC networks ${\bar{N}}_{1}$ and ${\bar{N}}_{2}$ have their leaves bijectively labeled on *S*, their construction from *N*_1_ and *N*_2_ is polynomial in the size of *N*_1_, *N*_2_, and *S*, and it is clear that *N*_1_≅*N*_2_ if, and only if, ${\bar{N}}_{1}≅{\bar{N}}_{2}$.

This shows that the isomorphism problem for TSTC networks with leaves bijectively labeled on *S* is polynomially equivalent to the isomorphism problem for TSTC networks with leaves injectively labeled on *S*, which is graph isomorphism-complete by Theorem 3.

## 4. Conclusion

We have proved that, unless the graph isomorphism problem belongs to P, there is no hope of defining a polynomially computable metric on the class of all TSTC networks on a set *S* of at least 3 taxa. It remains open the problem of defining polynomially computable metrics on the class of all TSTC networks on a given set *S* with all their hybrid nodes of in-degree bounded by some *d* ∈ *ℕ*. When *d* = 2, the *μ*-distance [7] and Nakhleh's *m* metric [8, 9] are such metrics, but they are no longer metrics for *d* = 3 (Figure  4 in [8]). Actually, we do not even know whether the isomorphism problem for TSTC networks on a given set *S* of taxa with globally bounded in-degree hybrid nodes (but without bounding the out-degree of the tree nodes; otherwise, Luks' theorem [19] would apply) is always in P, but we conjecture that this is the case.

## References

[1] Doolittle WF (1999). Phylogenetic classification and the universal tree. *Science*.

[2] Moret BME, Nakhleh L, Warnow T (2004). Phylogenetic networks: modeling, reconstructibility, and accuracy. *IEEE/ACM Transactions on Computational Biology and Bioinformatics*.

[3] Nakhleh L, Sun J, Warnow T, Linder CR, Moret BME, Tholse A (2003). Towards the development of computational tools for evaluating phylogenetic network reconstruction methods. *Proceedings of the 8th Pacific Symposium on Biocomputing*.

[4] Jin G, Nakhleh L, Snir S, Tuller T (2006). Maximum likelihood of phylogenetic networks. *Bioinformatics*.

[5] Jin G, Nakhleh L, Snir S, Tuller T (2007). Efficient parsimony-based methods for phylogenetic network reconstruction. *Bioinformatics*.

[6] Cardona G, Llabrés M, Rosselló F (2010). Two results on distances for phylogenetic networks. *Advances in Bioinformatics*.

[7] Cardona G, Llabrés M, Rosselló F, Valiente G (2008). A distance metric for a class of tree-sibling phylogenetic networks. *Bioinformatics*.

[8] Cardona G, Llabrès M, Rossellò F, Valiente G (2009). On Nakhleh’s metric for reduced phylogenetic networks. *IEEE/ACM Transactions on Computational Biology and Bioinformatics*.

[9] Nakhleh L (2010). A metric on the space of reduced phylogenetic networks. *IEEE/ACM Transactions on Computational Biology and Bioinformatics*.

[10] Nakhleh L (2004). *Phylogenetic networks [Ph.D. thesis]*.

[11] Cardona G, Llabrés M, Rosselló F, Valiente G (2009). Metrics for phylogenetic networks i: generalizations of the robinson-foulds metric. *IEEE/ACM Transactions on Computational Biology and Bioinformatics*.

[12] Cardona G, Llabrés M, Rosselió F, Valiente G (2009). Metrics for phylogenetic networks ii: nodal and triplets metrics. *IEEE/ACM Transactions on Computational Biology and Bioinformatics*.

[13] Goldberg M, Gross JL, Yellen J (2003). The graph isomorphism problem. *Handbook of Graph Theory*.

[14] Köbler J, Schöning U, Torán J (1993). *The Graph Isomorphism Problem: Its Structural Complexity*.

[15] Homann CM (1982). *Group-Theoretic Algorithms and Graph Isomorphism*.

[16] Baroni M, Semple C, Steel M (2006). Hybrids in real time. *Systematic Biology*.

[17] Cardona G, Rosselló F, Valiente G (2008). Tripartitions do not always discriminate phylogenetic networks. *Mathematical Biosciences*.

[18] Zemlyachenko VN, Korneenko NM, Tyshkevich RI (1985). Graph isomorphism problem. *Journal of Soviet Mathematics*.

[19] Luks EM (1982). Isomorphism of graphs of bounded valence can be tested in polynomial time. *Journal of Computer and System Sciences*.

